# A systematic review of simulation methods applied to cancer care services

**DOI:** 10.1080/20476965.2024.2322451

**Published:** 2024-03-01

**Authors:** Amalia Gjerloev, Sonya Crowe, Christina Pagel, Yogini Jani, Luca Grieco

**Affiliations:** a Clinical Operational Research Unit, Department of Mathematics, University College London, London, UK; b Centre for Medicines Optimisation Research & Education, UCLH NHS Foundation Trust & UCL School of Pharmacy, University College London, London, UK

**Keywords:** Systematic review, operational research, cancer care services, simulation

## Abstract

There is significant potential for Operational Research to support improvements in care services for cancer patients. In this systematic review, we examine computer simulation techniques used in supporting hospital-based cancer care, the type of problems addressed, the quality of the model and implementation, and the impact on patients. We identified 51 papers distributed between four problem types: patient flow/pathway modelling, scheduling, cost analysis, and resource allocation. Discrete Event Simulation was the most common simulation technique. Nearly two-thirds of the papers involved some form of engagement with clinicians or hospital managers: studies that did not reported fewer successful implementations. We discuss the reported benefits and limitations of applying simulation techniques to cancer care. Papers often highlighted opportunities to reduce hospital costs or waiting times, while a common limitation was a lack of, or limited, data. Stakeholder involvement throughout the project may mitigate obstacles and result in lasting policy changes.

## 1. Introduction

### 1.1. Cancer diagnosis and treatment is costly and Covid-19 disrupted services

In 2013, it was estimated that a quarter of a million people would be diagnosed with some form of cancer every year in the United Kingdom, leading to a yearly cost to society of approximately £12.8 billion (Luengo-Fernandez et al., 2013). In 2019, this estimate increased to a third of a million people (World Cancer Research Fund, 2023). The annual cost estimate included costs associated with premature deaths, time off work, medications, inpatient care, and outpatient care. In the United States, the cost of cancer care is increasing more than any other healthcare sector (Laviana et al., 2020), with the cost of medical services and oral prescription drugs expected to increase to $246 billion nationally by 2030 (Mariotto et al., 2020). Globally, it is becoming increasingly difficult to provide affordable and accessible cancer care (Sullivan et al., 2011). The COVID-19 pandemic severely disrupted cancer services, forcing hospitals to reorganise, adapt to new safety measures, and deal with cancellations of appointments, tests, and treatments (Chen-See, 2020; Edge et al., 2021). As a result, many patients are delayed in receiving diagnosis and treatment (Patt et al., 2020; Richards et al., 2020).

### 1.2. Timely care is important particularly for cancer

The process from referral to diagnosis and then treatment for cancer patients is called a care pathway. This process includes appointments, diagnostic tests and multidisciplinary team meetings, until a treatment plan is decided and started or the patient is discharged. In most cancer care services, reducing the time from diagnosis to treatment has been shown to improve the quality of life and survival rate of patients (Neal et al., 2015). For this reason, the times from referral to diagnosis and treatment are important metrics for tracking the quality of care. In the UK, the National Health Service (NHS) has set national target times that hospitals are expected to meet for patients with suspected cancer (NHS, 2022). These include a maximum of 62 days from initial referral to first treatment, and in England a maximum of two weeks from initial referral to the patient’s first appointment or diagnostic test, and 28 days from referral to diagnosis.

### 1.3. Cancer pathways must be rapid

Given that cancer pathways typically operate on short timelines to enable earlier diagnosis and treatment, testing out potential improvement using computer simulation can be much faster than implementing and evaluating all improvements in practice (Saville et al., 2019). In particular, simulation techniques have been used for a long time in healthcare (Fetter & Thompson, 1965) and are well suited for healthcare decision making (Mielczarek, 2016). The adoption of simulation by clinical decision makers has historically been low (Brailsford et al., 2009), although there are emerging examples of successfully implementing OR stochastic simulation projects with significant improvements in patient care (M. W. Carter & Busby, 2023, see references therein).

### 1.4. Our review aims to …

We conducted a systematic review to explore how computer simulation techniques have been used to analyse cancer care services across the referral-to-treatment pathway, with a focus on studies that could directly inform operational decisions. We exclude papers focused on cancer screening or prevention with the intention of assessing care pathways from referral through diagnosis (see Bespalov et al., 2021 for a review of simulation used for cancer screening). We aimed to:

1. Identify which OR simulation techniques have been used in cancer care services, how they have been applied, and the types of problems they have been used to address
2. Assess the quality of the models and their implementation, if applicable
3. Characterise any impact the modelling had on operational decisions made by clinical teams
4. Assess the level of engagement the simulation modellers had with clinical teams
5. Provide a searchable database of papers

In addressing these aims, we will support future research by highlighting how simulation has been used to date and identifying key factors for successfully informing improvements to cancer services in practice. In the following text, we present the review search and selection process in Section 2, and describe the reviewed papers along the dimensions of the five goals above in Section 3. In Section 4, we discuss the findings from the review, including limitations of the studies reviewed and areas for future research, and in section 5 we provide some concluding remarks.

## 2. Methods

The review methods we adopted conform to the guidelines for a systematic review outlined by Gough et al. (2012). Searches of PubMed, Scopus, and Web of Science were conducted to identify peer-reviewed papers published before May 2023 that contained the term “cancer” anywhere in the text, at least one simulation term and “topic term” in the abstract/title/keywords, and no “medical terms” in the title/keywords: Table 2 lists the search terms, which were informed by similar literature reviews (Aspland et al., 2021; Palmer et al., 2017; Saville et al., 2019). The medical terms and problem types were identified after running an initial search using only the cancer term and simulation terms. Four problem types were defined and determined the topic terms (Table 1). Our review protocol is registered on the International Prospective Register of Systematic Reviews (PROSPERO – CRD42021285893).

**Table 1.** Definitions of the problem type.

Problem Type	Definition
Patient Flow/Pathway Modelling	Papers that aimed to map and model the patient flow along a patient’s disease pathway, or map a workflow
Scheduling	Papers whose focus was on improving cancer service scheduling or analysing scheduling strategies
Resource Allocation	Papers that focused on the assignment or distribution of resources, e.g., hospitals beds, scanners, doctors/nurses
Cost Analysis	Papers whose metrics include financial costs related to cancer services

**Table 2.** Search logic used in literature searches.

	Cancer term	Simulation term	Topic term	Medical term
Search space	Abstract/Title/Keywords	Abstract/Title/Keywords	Title/Keywords	Title/Keywords
Search term	Cancer	Agent-Based	Appointment	Biolog*
		Agent Based	Bed occupancy	Biomarker*
		Computer simulation	Bottleneck	Biophysical*
		DES	Capacit*	Cell*
		Discrete event simulation	Capacity allocation	Clinical trial*
		Discrete-Event Simulation	Capacity management	DNA
		Monte Carlo	Capacity planning	Dosimetr*
		Simulation	Cost-benefit	Epidemiolog
		Simulation model	Cost-effective*	Genom*
		Stochastic model*	Cost-util*	Genotyp*
		Stochastic processes	Cost*	Molecul*
		Stochastic analysis	Economic evaluation	Oncology trial*
		System dynamics	Economic*	Patholog*
		Visual simulation	Healthcare OR	Prevent*
			Operational performance	Protein
			Operational problem	Screen*
			Operational research	Signal*
			Operations research	Silico*
			Pathway	
			Patient care	
			Patient flow	
			Patient route	
			Patient throughput	
			Planning policy	
			Planning process	
			Process flow	
			Queue*	
			Queuing	
			Resource allocation	
			Resource management	
			Resource planning	
			Resource util*	
			Schedul*	
			Service improvement	
			wait* time	
			Waiting list	

Table 3 gives the data extraction template we applied to each included paper, which we based on the STRESS guidelines for reporting on simulation studies (Monks et al., 2019). To pass the “STRESS-test” as defined by Monks et al., a simulation paper must explain the objectives, logic, data, experimentation, and implementation of their project.

**Table 3.** Data extraction parameters.

Field	Description
Techniques	Modelling approach/solution method implemented (e.g., Discrete Event Simulation or Agent-Based).
Model objective	Goal of the model (e.g., simulate waiting times, calculate costs associated with screening strategies).
Project objective	Goal of the project (e.g., optimise patient flow along a pathway, improve efficiency of screening strategies).
Metrics	What metrics were measured to identify the success of the simulation?
Software	What software was used to build the simulation?
Data set	What data were used to parameterise the model?
Constraints	What were the parameters and constraints imposed on the model?
Limitations	What were the limitations of the model and project?
Benefits of the approach/methodology	What was improved by the project?
Summary of the pathway	What portion of the patient pathway was modelled (e.g., referral to treatment)?
Cancer type	Which cancer/treatment did the project focus on?
Operational engagement	Were clinicians or operational managers involved in the project and to what extent?
Implementation	Were the suggested interventions used in practice by the hospital?
Suggested changes	What interventions or changes in policy were suggested by the paper?

To reduce the risk of bias and validate the results of the search, the quality of the papers was assessed using a modified version of the critical appraisal checklist used by Fone et al. (2003) and Neal et al. (2015). Our quality assurance checklist included the following items: the objective of the study, the success of the implementation of the project, the engagement of the client/operational team, the sample size or the data set used to parameterise the model, constraints imposed on the model, and the limitations of the study. These items were incorporated into the list of data to be extracted (Table 3).

Papers were deemed out of scope and removed based on the exclusion reasons set out in Table 4, initially upon review of titles and keywords, then abstracts, then full text. Finally, 10% of the papers were randomly selected and reviewed by a second reviewer. Any disputed or contentious papers from the full-text reading were further analysed and discussed with the second reviewer to determine whether to include these papers. This edge case review served as a quality control check and ensured that there were no systematic issues with the screening and data extraction process.

**Table 4.** Exclusion criteria for assessing the papers.

Exclusion reasons
Does not solve a cancer care problem. For example:
(a) Does not focus on cancer care services; exclude papers that are simply “inspired” by cancer centres but do not address cancer care services anywhere else in the paper
(b) Was not within a healthcare context, e.g., identifying online spending habits of cancer patients.
(c) The focus is not on improving cancer care services or would not drive a change in hospital operational decisions.
(d) The primary focus of the paper or model was on the evolution of the disease or progression of health states, e.g., tumour growth models.
(e) Focuses on the improvement of cancer treatment techniques, e.g., intensity-modulated radiation therapy, using clinical metrics, e.g., average dose given
(f) The focus is on cancer prevention or screening, e.g., the effect of smoking, heavy metals in river water, obesity, suboptimal breast feeding on cancer risk
(g) Focuses on drug administration techniques.
(2) Does not use a computer simulation technique. For example:
(a) The techniques used only include integer programming and simplex algorithms, data mining techniques, machine learning, or mixed-integer linear programming
(b) The simulation technique was only used to conduct sensitivity analysis and was not the principal simulation technique.
(3) Is a review of OR techniques or a literature review
(4) Unable to obtain the full text or an English language version.

The final set of papers were divided into the predefined categories based on the type of problem addressed, simulation methods, software, cancer/treatment focus, and the type of operational engagement (see below). Papers that addressed more than one different problem types were included in each relevant problem group.

We defined four distinct types of operational engagement:

- **None**: there was no mention of engagement with operational managers/clinicians
- **Parameter estimation/model assumptions**: there was some mention that operational managers/clinicians were consulted when creating the model, parameterising the model, or during data collection
- **Model validation**: there was some mention of operational managers/clinicians contributing to the model validation, and determining that the initial results were representative of real life
- **Intervention/policy changes**: there was some mention of engaging with operational managers/clinicians when determining the final interventions or policy changes

Additionally, we define three levels of implementation as the following:

- **Not mentioned**: no information was provided about implementation, or it was stated that no changes were being made by the hospital
- **Currently being evaluated**: the suggested changes were being assessed or trialled by the hospital at the time the paper was published.
- **Implemented**: the suggested interventions were used in practice by the hospital

Implementations and suggested changes can be categorised as hospital-level changes, organisational level changes, and system-level changes. We define hospital-level changes as interventions that would be implemented at one hospital, such as increasing the number of available nurses at an outpatient clinic. Organisation-level changes would be implemented across a group of hospitals within a trust or organisation, and system-level changes would be made at many organisations or a geographical region.

## 3. Results

**Figure 1.**
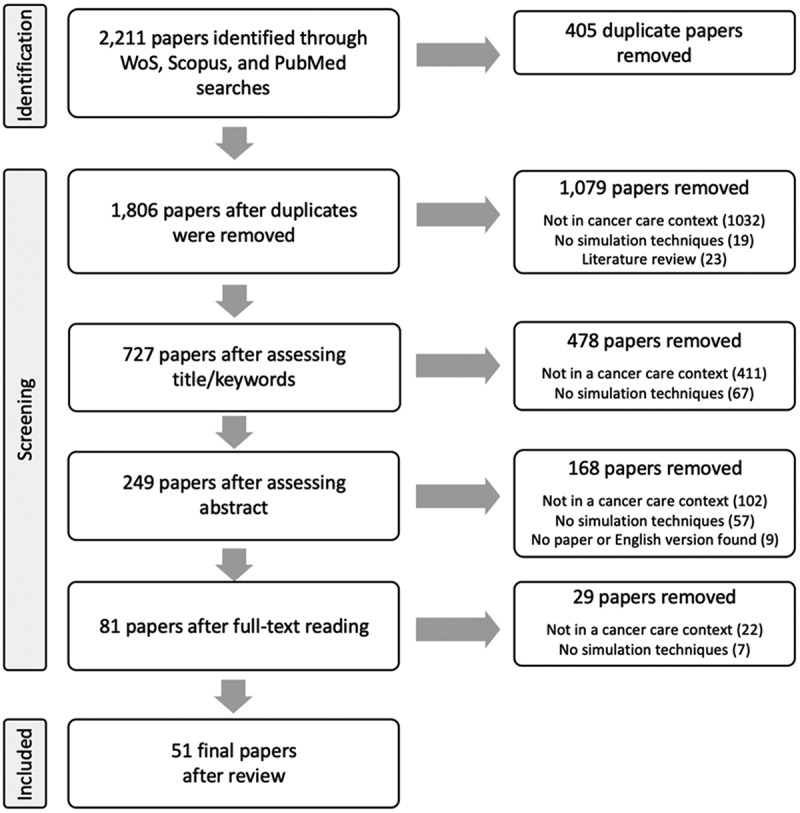
Flow diagram illustrating the number of papers that were excluded at each step of the data extraction.

The initial search identified 2,211 papers. After the removal of duplicates, 1,806 titles, keywords, and abstracts were screened, resulting in 249 papers for full text review, of which 51 were included for the review (Figure 1). The results are presented by describing the study characteristics, and synthesising the level of operational engagement, the reported implementation and service changes, and the type of problem addressed (patient flow, scheduling, resource allocation, etc.).

Studies were commonly published in healthcare management, systems engineering, and decision-making journals. The largest group of papers focused on patient flow, with smaller sets of studies dealing with the other three categories of interest. Twenty per cent of the selected papers addressed two problem types.

**Figure 2.**
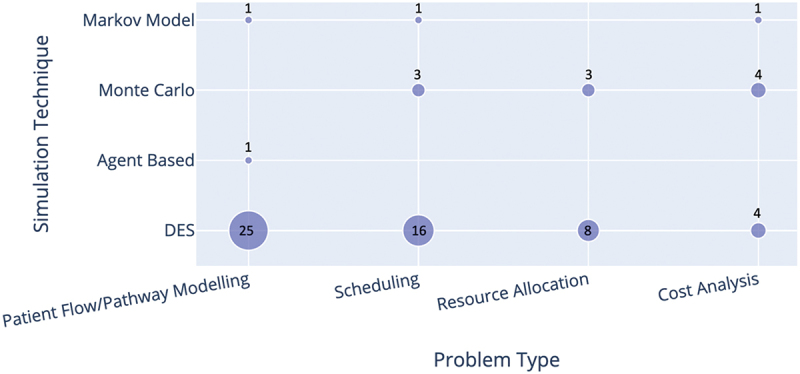
Cross analysis of the simulation techniques and problem type. The number of studies is shown by/in each circle. Papers that used multiple simulation techniques were included in each category.

**Figure 3.**
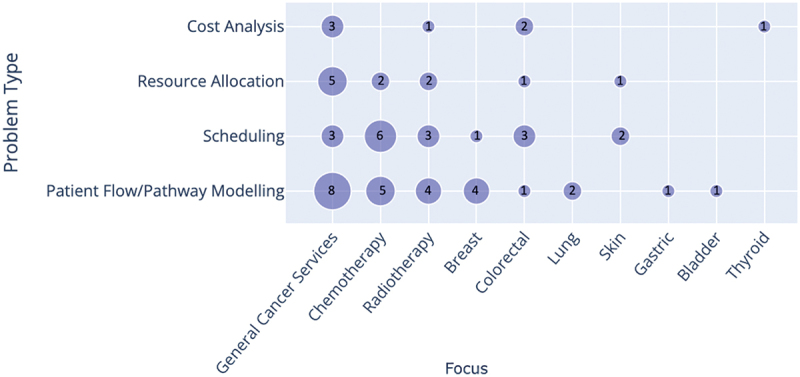
Cross analysis of the cancer/treatments addressed by the paper and the problem type. The number of studies is shown by/in each circle.

Figures 2 and 3 classify papers in each problem type category by cancer/service type and by stochastic simulation techniques, respectively. While there were some studies of specific cancer types (mainly breast and colorectal cancer), most publications were related to diagnostic/treatment services in general, or to chemotherapy/radiotherapy settings. Discrete Event Simulation (DES) was the most used simulation technique (more than 80% of the selected publications), especially for models dealing with patient flow across the entire pathway from diagnostic/treatment services to appointment scheduling and resource allocation. Forty-two papers in total utilised DES in their study, with only three (6%) papers using another simulation technique alongside DES (Elliott et al. (2019), Martin et al. (2020), and Blakely et al. (2015)). Other techniques used are Markovian Simulation, System Dynamics Monte Carlo Simulation, and Agent-Based Simulation.

### 3.1. Operational engagement

**Figure 4.**
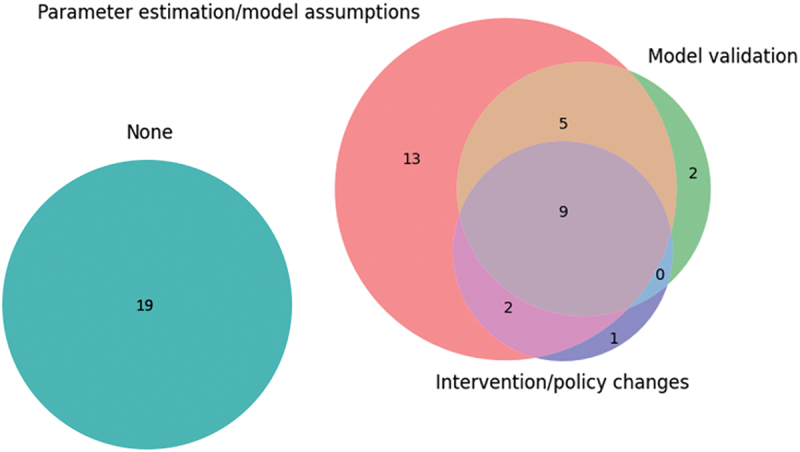
Frequency of papers within each type of operational engagement: parameter estimation/model assumptions (red), model validation (green), intervention/policy changes (dark blue), none (turquoise).

We defined operational engagement as the consultation or involvement of operational managers, clinicians, or healthcare professionals. The extent of operational engagement among the papers is shown in Figure 4. Nine (17%) studies mentioned involving clinicians or operational managers along all the steps of creating and parameterising the model, validating the model, and determining appropriate interventions or policy changes. Thirty-one studies reported consulting healthcare professionals or operational managers when designing their experiment and 19 papers mentioned operational engagement when validating their model or defining feasible interventions. Nineteen (34%) of the papers did not mention operational engagement at all and instead derived their models from other sources, e.g., peer-reviewed papers. Compared to the cost analysis, resource allocation, and scheduling papers, pathway modelling papers reported more engagement with 73% of the papers mentioning some form of hospital management or clinician engagement.

### 3.2. Implementation

**Figure 5.**
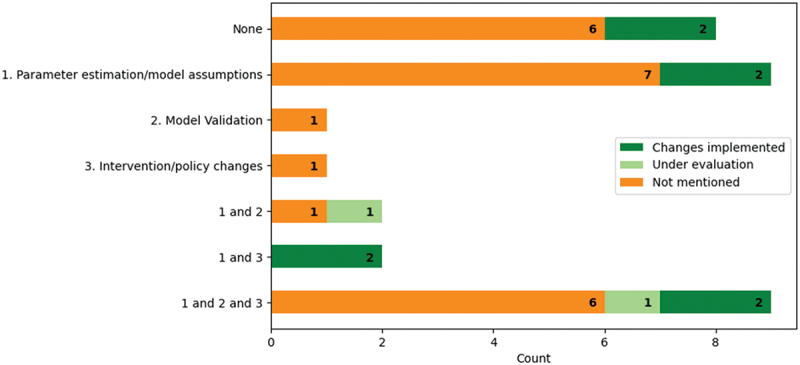
Frequency of papers with implementations within each type of operational engagement mentioned.

Eight (16%) out of 51 papers mentioned successful implementations: four for pathway modelling, two for scheduling, and two papers addressing pathway modelling and resource allocation/scheduling problems. A further two of the 51 papers reported interventions being evaluated by the hospital at the time of writing. No papers reported unsuccessful implementations. Eighteen papers did not specify that their objective was to improve services in practice. Of the remaining 33 papers, 23 (70%) reported involving clinicians or operational managers in parameter estimations/modelling assumptions. Twenty-four per cent of the 32 studies whose aim was to improve services indicated that their suggested interventions were implemented (Figure 5). Successful implementations were most common among projects that involved clinicians both in determining parameters and determining feasible interventions or potential policy changes.

The implementation of new policies was low in studies that addressed problems related to resource allocation or cost analysis. Two of 11 resource allocation papers stated that new policies or recommendations are being considered by senior management or were trialled in a pilot study (Romero et al., 2013; Santibáñez et al., 2009). Successful implementations were not mentioned in any of the seven cost analysis papers and only three papers indicated engaging with operational managers or clinicians. Seven of the eight successful implementation papers had clinical co-authors and one heavily involved the voice of the patient in their study (Arafeh et al., 2014).

**Figure 6.**
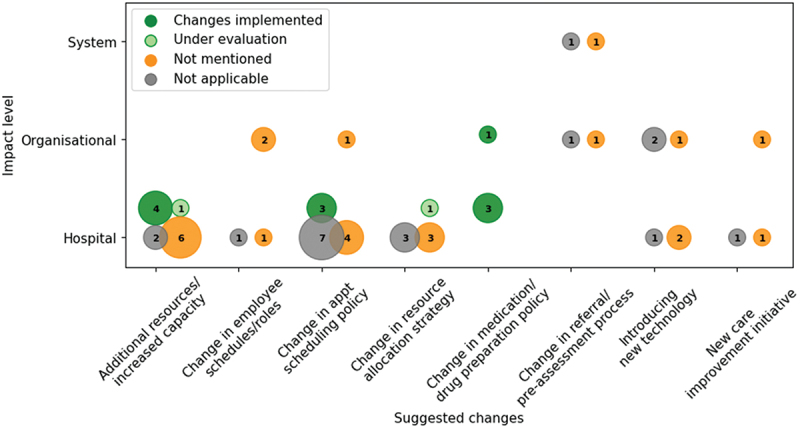
Frequency of suggested changes found in the papers and the impact level of the changes: hospital-level, organisational-level, or the system-level. The number indicates the count of papers. Colours indicate whether those changes were implemented, are under evaluation, were not described as implemented, or implementation is not applicable.

The recommended changes to cancer services made by the papers, the impact level of the change, and implementation status are shown in Figure 6. The majority of suggested changes were at the hospital-level.

We define eight categories of suggested changes. Some of these categories inherently fall into one of the impact levels, e.g., a change in the cancer referral process would affect general practitioners across a system or organisation. Some papers suggested a combination of changes. The most common suggestions include increasing the capacity or adding resources to a clinic, proposing a change in appointment scheduling policies (such as changing the arrival pattern of patients to fit nursing schedules (Ahmed et al., 2011)), and new resource allocation strategies (such as changing the ratio of procedure rooms to endoscopists (B. Berg et al., 2010)). Organisation-level suggestions included introducing new technology to the hospital or organisation, such as using a real-time location system (Kang & Haswell, 2020) and running a new care improvement initiative (Round et al., 2014).

Thirteen papers suggested adding resources or increasing the capacity, all of which were at the hospital level. Six of the 15 papers suggesting changes to scheduling policies were novel scheduling algorithms. The most implemented suggestions were at the hospital level and include an increase in capacity, changes in appointment scheduling policy, or changes in medication or drug preparation policies.

### 3.3. Patient Flow/Pathway modelling

A summary of the 26 papers addressing patient flow/pathway modelling problems is given in Table 5.

**Table 5.** Summary of papers that address problems related to patient flow/pathway modelling.

Reference	Simulation Method	Project focus	Project objective	Software used	Metrics
Arafeh et al. (2014)	DES	All (pharmacy modelling)	Reduce patients’ waiting time in an outpatient pharmacy located in a cancer treatment hospital using a six Sigma process	Microsoft Visio, Promodel, EasyFit	Prescription preparation time
Holm et al. (2017)	DES	All (surgical unit)	Investigate effect of different interventions on patient flow and resource utilisation	FlexSim Healthcare	Number of weekly surgery slots, proportion of finished surgeries within opening hours
Kang and Haswell (2020)	DES	All (oncology scheduling)	Understand the impact of a real-time locating system on oncology clinic and assess potential changes to staff scheduling and care process	FlexSim Healthcare	Wait time, length of stay, proportion of patients waiting more than 30min
McKinley et al. (2020)	DES	All (children’s care)	Evaluate the impact on system flow of a time-specific quality improvement protocol for children with cancer and central venous catheters who present to the paediatric ED with fever	Arena	Wait time, length of stay
Rejeb et al. (2018)	DES	All (cancer treatment)	Assess the impact of a Health Information System on the patient pathway	Arena	Time to discharge of the patient, avg. duration to access information, waiting time, resource utilisations, resource costs, service quality
Santibáñez et al. (2009)	DES	All (ambulatory care)	Reduce wait times and address significant challenges regarding the use of space and resources in an ambulatory care unit	Arena	Wait time, clinic end time, and physician idle time
Singh et al. (2022)	DES	All (outpatient department)	Optimise and predict the resource requirements for an outpatient department	FlexSim Healthcare	Resource utilisation, wait time, total throughput
Suss et al. (2018)	DES	All (outpatient clinic)	Improve flow of patients through outpatient oncology clinic, focusing on the steps upstream of the infusion step	Arena	Wait times, pharmacy resource utilisation
Ahmed et al. (2011)	DES	Chemotherapy	Improve the performance of a chemotherapy treatment unit by increasing throughput and reducing average patient waiting time	Arena	Average num. of patients served, wait time, resource utilisation
Alvarado et al. (2018)	DES	Chemotherapy	Evaluate patient and resource scheduling rules for chemotherapy patients	DevsJava	Patient throughput, resource and nurse utilisation
Bernatchou et al. (2017)	DES	Chemotherapy	Evaluate patient flow performance through the outpatient chemotherapy unit	Arena	Wait times, resource utilisation
Lamé et al. (2020)	DES	Chemotherapy	Map the care process to improve patient flow in a chemotherapy unit and chemotherapy preparation pharmacy	ARENA	Wait time
Lu et al. (2012)	DES	Chemotherapy	Optimise treatment delivery and reduce patients’ average waiting time when receiving chemotherapy for different dose administration strategies	Simul8	Drug preparation and delivery time
Babashov et al. (2017)	DES	Radiotherapy	Reduce wait times and determine bottlenecks in the radiotherapy planning process	Simul8	Mean ready-to-treat to treatment time, percentage of patients treated within target time, resource utilisation
Boonmee et al. (2020)	DES	Radiotherapy	Improve patient flow and identify bottlenecks in radiotherapy services	Arena	Number of patients, time spent in a room, operation time, resource utilisation
Miranda and Miranda (2021)	DES	Radiotherapy	Reduce wait times and improve patient throughput along the radiotherapy pathway	Arena	Wait time, patient throughput, entrance time of technicians, exit time of the last patient
Vieira et al. (2019)	DES	Radiotherapy	Assess the impact of using pull and push scheduling strategies in waiting time between referral and first treatment	Tecnomatix Siemens Plant Simulation	Wait time, percentage of patients treated within target time
Camg ¨oz Akda ˘g and Arsoy-Ilikan (2018)	DES	Breast	Decrease patient wait times in a breast cancer centre	Arena	Wait times
Coelli et al. (2007)	DES	Breast	Understand the global assessment of patient flow, equipment utilisation, and personnel needs in a mammography clinic	Promodel	Length of stay, physician/technician utilisation
Smith et al. (2022)	DES	Breast	Assess the impact of a risk prediction tool on flow of patients through a clinic	Simul8	Throughput time, proportion of patient journeys exceeding target times, percent of patient receiving immediate referral
Walker et al. (2016)	Bootstrap resampling with simulations	Breast	Identify how additional resources could be best utilised to reduce delays	R	Throughput time, proportion of patient journeys exceeding target times
Elliott et al. (2019)	DES, Agent-based	Colorectal	Assess the impact of a risk prediction tool on patient bookings and wait times for colonoscopies	AnyLogic	Wait times, hospital costs
T. J. England et al. (2021)	DES	Lung	Identify areas for improving patient throughput times in the lung cancer pathway	Simul8	Average time in the system, percentage of patients treated within target time
T. England et al. (2021)	DES	Lung	Assess the impact of a modified lung cancer pathway on patient flow through the system	Simul8	Average time spent in the system, percentage of patients treated within target time
H. E. Carter et al. (2019)	DES	Gastric	Reduce wait times along the along endoscopic pathway and forecast the impacts of future endoscopic demand	AnyLogic	QALY, incremental cost-effectiveness ratio
Chalk et al. (2019)	DES	Bladder	Model patient flow along bladder cancer pathway to identify bottlenecks and reduce patient delays	Simul8	Average time spent in the system

#### 3.3.1. Types of pathways modelled

Modelling the flow of patients along a pathway was the most common problem type, with 26 (51%) papers, of which 25 papers used DES (Table 5). The modelled pathways followed the patient from hospital arrival to treatment to discharge or from initial referral to diagnostic appointments to treatment. Five papers investigated drug preparation pathways, which included the steps between receiving the prescription to drug preparation to administering the drug (Alvarado et al., 2018; Arafeh et al., 2014; Kang & Haswell, 2020; Lu et al., 2012).

#### 3.3.2. Metrics used

Half of the papers measured the average wait time experienced by patients in the pathway to evaluate how the system behaves under various what-if scenarios. Other common metrics included the proportion of time that resources are idle (service utilisation) and the average time a patient spends in the system (throughput time).

#### 3.3.3. Types of investigations undertaken

Papers in this section identified bottlenecks, compared new decision-making strategies or proposed protocols, and holistically assessed the pathway. One paper demonstrated how to apply the six Sigma methodology in combination with DES to analyse pathway characteristics and the efficiency of a cancer centre (Arafeh et al., 2014). The study focused on the voice of the customer and conducted a detailed root cause analysis. The voice of the customer, in this case patients, is another facet of patient care and can be used to improve healthcare services (Al-Abri & Al-Balushi, 2014). Another paper had a similar goal of illustrating how a technique could be used in a healthcare services project (Kang & Haswell, 2020). This paper showed how using data from a real-time location system could be used along with electronic health record data to capture the detailed aspects of patient care, and how this could be used with DES to smoothly analyse patient flow.

#### 3.3.4. Papers also addressing resource allocation

Three papers addressed the allocation of resources in addition to improving patient flow (Holm et al., 2017; Santibáñez et al., 2009; Singh et al., 2022). The paper by Singh et al. (2022) ran five what-if scenarios to determine the optimal time between appointments and distribution of receptionists, rooms, and technicians at an outpatient clinic in India. Holm et al. (2017) used soft systems methodologies and DES to identify problem areas along the patient pathway in a surgical unit. They evaluated the impact of five feasible interventions and developed an algorithm for finding an optimal scheduling of planned surgeries. Santibáñez et al. (2009) simulated various strategies for improving ambulatory care in a cancer centre, including how rooms are allocated to patients and varying the clinic start time.

#### 3.3.5. Software used

ARENA and Simul8 were the most popular software packages, with 10 and six papers using each respectively. Three studies used FlexSim Healthcare software, two used AnyLogic, and the remaining five studies used different packages. This is consistent with the work of Pereira et al. (2011), who found that ARENA and Simul8 to be the “most used” and “best” software packages for DES. Almagooshi (2015) found that DES models are often programmed using ARENA or other high-level software packages rather than programming languages such as Python or C++.

#### 3.3.6. Operational engagement

Operational engagement was typically mentioned in only one aspect (Figure 4). Eighteen (69%) of the 26 pathway modelling papers involved operational managers or clinicians in their parameter estimations or model development, with 12 (46%) papers also mentioning engagement in validating their model or determining feasible interventions/policy changes. Additionally, five projects reported successful implementation (Ahmed et al., 2011; Arafeh et al., 2014; H. E. Carter et al., 2019; Chalk et al., 2019; Lu et al., 2012) and two mentioned that their suggestions and interventions were under discussion for being implemented (Lamé et al., 2020; Santibáñez et al., 2009). One example of a successful implementation can be seen in the paper by Lu et al. (2012), where the hospital saw a 50% decrease in the delivery time of antineoplastic medication, a chemotherapy medication, consistent with the scale of benefit from their simulation.

#### 3.3.7. Changes suggested

The changes recommended for implementation were generally specific to the hospitals. These changes included increasing the number of employees, increasing the opening hours of a clinic, or creating new appointment scheduling policies and resource allocation strategies. Some papers provided results that were generalisable to other organisations, such as novel simulations, scheduling algorithms, or care management strategies. For example, Lamé et al. combined DES and soft systems methodologies to model a chemotherapy outpatient unit. They found that creating a specific nursing role dedicating to reaching out to chemotherapy patients prior to treatment reduced wait times and increased revenue (2020). Approximately a quarter of the papers presented organisational or system-level changes. These included implementing real time location services (Kang & Haswell, 2020) and utilising a new risk assessment tool (Elliott et al., 2019; Smith et al., 2022).

### 3.4. Scheduling

A summary of the 18 papers addressing scheduling problems is given in Table 6.

**Table 6.** Summary of papers that address problems related to scheduling.

Reference	Simulation method	Project focus	Project objective	Software used	Metrics
Keshtzari and Norman (2022)	DES	All (outpatient clinics)	Identify the required clinic capacity to give timely appointments for cancer patients	Simio	Mean wait time, capacity and patient deviation from treatment plans
Ma et al. (2016)	DES	All (oncology scheduling)	Assess appointment scheduling for new patient consultations	Java, R	Weekly number of add-on consults, the percentage of patients booked within target times, appointment slot utilisation
Ogulata et al. (2009)	DES	All (oncology scheduling)	Calculate the impact of varying the maximum wait times, arrival rates, and slack capacity on delays and accepted/unaccepted patients	SIMAN	Percentage of unaccepted patients, treatment delay times
Ahmed et al. (2011)	DES	Chemotherapy	Improve the performance of a chemotherapy treatment unit by increasing throughput and reducing average patient waiting time	Arena	Average num. of patients served, wait time, resource utilisation
Hadid et al. (2022)	Stochastic Discrete Simulation-Based Multi-Objective Optimization, Monte Carlo	Chemotherapy	Improve scheduling of outpatient chemotherapy appointments and minimise staff overtime and the length of stay of patients	Minitab, OptQuest	Appt make span and patient length of stay (make span), and overtime of resources, utilisation rates, wait times
Heshmat and Eltawil (2021)	DES	Chemotherapy	Find the optimal chemotherapy schedule and patient appointment schedules	ARENA	Wait times
Masselink et al. (2012)	DES	Chemotherapy	Calculate average waiting time for chemotherapy and pharmacy costs under different scheduling strategies	MATLAB	Wait times, cost per day
Liu et al. (2019)	DES	Chemotherapy	Measure demand and treatment capacity to provide timely and quality care to chemotherapy patients	SIMIO	Clinic overtime, patient wait times, nurse direct care time, number of pharmacy preparations
Slocum et al. (2021)	DES	Chemotherapy	Reduce patient wait times and nurse overtime requirements in outpatient chemotherapy operations	Microsoft Excel, VBA	Wait times, average nurse overtime
Bauza and Chow (2019)	Monte Carlo	Radiotherapy services	Improve scheduling for on-call physicists and decrease the number of overworked physicists	MATLAB	Work shift costs
Werker et al. (2009)	DES	Radiotherapy services	Simulate time taken to conduct radiation therapy planning	ARENA	Planning time
Vieira et al. (2019)	DES	Radiotherapy services	Assess the impact of using pull and push scheduling strategies in waiting time between referral and first treatment	Tecnomatix Siemens Plant Simulation	Wait time, percentage of patients treated within target time
van de Vrugt et al. (2017)	DES	Breast	Evaluate scheduling strategies to provide 90% of patients with an appointment within a week	Microsoft Excel, C++ DES Mersenne Twister	Wait time, time to diagnosis, access time
B. P. Berg et al. (2013)	DES	Colorectal	Calculate the cost of no-shows and evaluate the effects of mitigation strategies as well as overbooking policies	ARENA	Net gain ($)
Kazemian et al. (2017)	DES	Colorectal	Simulate patients of different acuity levels moving along surgery pathway to evaluate scheduling policies	MATLAB	Average overtime per day
Martin et al. (2020)	DES, Monte Carlo	Colorectal	Inform clinics on benefits of various scheduling policies	C++ Visual Studio	Wait time, resource idle time, resource overtime
Burns et al. (2022)	DES	Skin	Simulate patient flow through a surgery clinic to assess scheduling strategies	ARENA	Wait times, patient throughput, clinic overtime
Romero et al. (2013)	DES	Skin	Measure and calculate advantages of average throughput time when implementing a one-stop-shop at a skin cancer clinic	ARENA, VBA, Microsoft Excel	Patient throughput time, number of patients treated with Excision/Photodynamic therapy

#### 3.4.1. Types of investigations undertaken

Scheduling problems include finding optimal appointment schedules, identifying strategies for alleviating overbooking policies, or assessing various scheduling strategies. For example, van de Vrugt et al. (2017) conducted a study of a breast cancer centre, the results of which uncovered hidden capacity problems and identified a scheduling strategy for reducing wait times. Another example is Kazemian et al. (2017), who identified appointment scheduling policies that would reduce the average daily operating room overtime.

#### 3.4.2. Metrics used

Patient waiting time, clinician or nurse overtime, and the total clinic throughput were commonly used metrics. One study quantified the gap between the demand and capacity of nurses by defining “Nurse Direct Care Time” (total patient one-on-one time spent by a nurse) (Liu et al., 2019). This study also defined the “Number of Pharmacy Preparations” (total number of drugs prepared by a pharmacy technician per patient appointment) to further assess the clinic’s workload. Another study considered the cost associated with no-shows, patients who fail to attend their appointment, and tested four scheduling strategies for reducing no-shows (B. P. Berg et al., 2013).

#### 3.4.3. DES Simulation papers

DES was used by 16 (89%) of the papers in this category. Two papers integrated linear programming with DES to find optimal schedules (Heshmat & Eltawil, 2021; Romero et al., 2013). For example, Heshmat and Eltawil used mixed integer programming to find the optimal number of nurses, pharmacists, and patients for a chemotherapy clinic schedule. DES was then used to calculate the waiting time, generate appointment schedules, and calculate the total completion times. The results of the DES model were used to analyse the optimal scheduling rules under various resource availability constraints.

#### 3.4.4. Other types of simulations used

Three papers used Monte Carlo simulations (Bauza & Chow, 2019; Hadid et al., 2022; Martin et al., 2020), with one paper using Monte Carlo simulations in combination with a stochastic discrete simulation-based multi-objective optimisation model to find the optimal scheduling policy for an outpatient chemotherapy clinic (Hadid et al., 2022). One project used Monte Carlo replications to create an automated scheduling system for on-call physicists working in a cancer centre (Bauza & Chow, 2019). Their proposed scheduling system would reduce the number of overworked physicists and mean shifts per person.

#### 3.4.5. Changes suggested

Recommended changes to appointment scheduling policies were typically specific to the collaborating hospital. For example, one paper suggested altering the overbooking policy and implementing a no-show intervention, and calculated the resulting financial gain (B. P. Berg et al., 2013). Other papers presented novel scheduling algorithms that could be implemented at other organisations (e.g., Ma et al. (2016); Ogulata et al. (2009); Hadid et al. (2022)).

#### 3.4.6. Operational engagement

Eleven out 18 (61%) studies mentioned operational engagement, with two mentioning involvement in all three aspects: intervention/policy changes, parameter estimation/model assumptions, and model validation. For example, Masselink et al. (2012) aimed to identify pharmacy costs under different chemotherapy scheduling strategies and found that reusing the most expensive medicines led to lower costs and waiting times. Staff contributed to the development of the model and the new protocol was implemented in a Dutch Chemotherapy Day Unit in 2009. van de Vrugt et al. (2017) recommended adding two extra slots for unscheduled appointments at a breast cancer clinic, a suggestion that was implemented and led to 92% of patients receiving their diagnosis within five days of their diagnosis. In this project, clinicians and clinical managers were involved in defining and validating the model and determining feasible interventions. In another project by (Ahmed et al., 2011), changing the arrival pattern of patients to match the nurses’ schedules led to the greatest improvement in the performance of a chemotherapy clinic. A scheduling template was created based on this information, which was implemented at the hospital, with intentions of rolling out the template to other hospitals and community cancer sites. Five papers only involved clinicians or operational managers in one aspect of their process.

### 3.5. Resource allocation

A summary of the 11 papers addressing allocation of resource problems is given in Table 7.

**Table 7.** Summary of papers that address problems related to resource allocation.

Reference	Simulation method	Project focus	Project objective	Software used	Metrics
Holm et al. (2017)	DES	All (surgical unit)	Investigate effect of different interventions on patient flow and resource utilisation	FlexSim Healthcare	Number of weekly surgery slots, proportion of finished surgeries within opening hours
Keshtzari and Norman (2022)	DES	All (outpatient clinics)	Identify the required clinic capacity to give timely appointments for cancer patients	Simio	Mean wait time, capacity and patient deviation from treatment plans
Santibáñez et al. (2009)	DES	All (ambulatory care)	Reduce wait times and address significant challenges regarding the use of space and resources in an ambulatory care unit	ARENA	Wait time, clinic end time, and physician idle time
Singh et al. (2022)	DES	All (outpatient department)	Optimise and predict the resource requirements for an outpatient department	Flexsim	Resource utilisation, wait time, total throughput
S. Thomas et al. (2001)	Monte Carlo	All (outpatient clinics)	Identify how much surplus capacity is needed to maintain low wait times for clinics in the UK	C++	Capacity, percentage of untreated patients
Baril et al. (2020)	DES	Chemotherapy	Optimise nurse workload in a chemotherapy workflow	ARENA	Nurse utilisation, average time tasks wait before being completed
Liu et al. (2019)	DES	Chemotherapy	Measure demand and treatment capacity to provide timely and quality care to chemotherapy patients	SIMIO	Clinic overtime, patient wait times, nurse direct care time, and number of pharmacy preparations
Bauza and Chow (2019)	Monte Carlo	Radiotherapy services	Improve scheduling for on-call physicists and decrease the number of overworked physicists	MATLAB	Work shift costs
S. J. Thomas (2003)	Monte Carlo	Radiotherapy services	Predict the number of linear accelerators required for radiotherapy treatment machines	C++	Percentage of patients starting treatment in less than days
B. Berg et al. (2010)	DES	Colorectal	Optimise resource allocation for colonoscopy screening in a colonoscopy suite	ARENA	Wait time, resource utilisation, turnover time
Romero et al. (2013)	DES	Skin	Measure and calculate advantages of average throughput time when implementing a one-stop-shop at a skin cancer clinic	ARENA, VBA, Microsoft Excel	Patient throughput time, number of patients treated with Excision/Photodynamic therapy

#### 3.5.1. Types of investigations undertaken and metrics used

11/51 (22%) papers focused on allocation of resources. DES was again the most common simulation technique (8/11, 73%). The papers that employed DES commonly looked at patient wait times along pathways, clinic overtime, and resource idle time (e.g., how long are physicians waiting between appointments or waiting for patients). The papers using DES varied from determining the optimal staffing levels of nurses, to simulating the benefits of implementing a one-stop-shop for treatment, to measuring the utilisation of resources (e.g., procedure rooms, endoscopists). In addition, four studies were carried out to examine the effectiveness of appointment scheduling policies in conjunction with the assessment of capacity planning and/or staffing assignments (Bauza & Chow, 2019; Keshtzari & Norman, 2022; Liu et al., 2019; Romero et al., 2013).

#### 3.5.2. Changes suggested

The changes suggested by the papers were generally changes in resource allocation strategies specific to the collaborating hospital, such as changing the number of full-time nurses (Liu et al., 2019) or increasing the number of clinic hours (Holm et al., 2017). Some papers presented generalisable changes that could be applied to other organisations, e.g., Thomas et al. introduced an equation for determining the capacity based on the referrals (2001).

#### 3.5.3. Implementations

No papers mentioned their findings being implemented in practice, although one paper mentioned successfully conducting an initial pilot study with promising results (Romero et al., 2013). In this paper, Romero et al. (2013) describes assembling a one-stop-shop within a Pathology department for skin cancer patients. Another paper stated their recommendations were proposed to senior management and were being considered for implementation at the time of writing (Santibáñez et al., 2009). A unique feature of their DES model was the simultaneous operation of independent clinics at a cancer centre. The results highlighted the importance of clinic start times and room allocations on patient wait times.

### 3.6. Cost analysis

A summary of the seven papers addressing scheduling problems is given in Table 8.

**Table 8.** Summary of papers that address problems related to cost analysis.

Reference	Simulation method	Project focus	Project objective	Software used	Metrics
Blakely et al. (2015)	Des, Monte Carlo	All (cancer care)	Determine the cost-utility of a cancer care coordinator intervention in stage III colon cancer	Tree Age Pro	Incremental cost-effectiveness ratio, QALYs, incremental costs per patient
Rejeb et al. (2018)	DES	All (cancer treatment)	Assess the impact of a Health Information System on the patient pathway	Arena	Time to discharge of the patient, avg. duration to access information, waiting time, resource utilisations, resource costs, service quality
Round et al. (2014)	Monte Carlo	All (hospice care)	Assess the cost effectiveness of a hospice based day therapy intervention for patients living “beyond” cancer	Not mentioned	Incremental cost-effectiveness ratio, QALYs
B. P. Berg et al. (2013)	DES	Colorectal	Calculate the cost of no-shows and evaluate the effects of mitigation strategies as well as overbooking policies	ARENA	Net gain ($) and cost of no-shows
Pilgrim et al. (2009)	DES	Colorectal	Identify cost effectiveness of different care strategies for bowel cancer patients	Simul8	QALYs gained, marginal costs
Price et al. (2022)	Monte Carlo	Radiotherapy	Assess the cost viability of a fully mobile radiation oncology clinic in rural areas	Python	Profit per year, patients seen per year
Heller et al. (2012)	Markov Model, Monte Carlo	Thyroid	Assess the economic impact of Fine-Needle Aspiration treatment for thyroid cancer patients	TreeAge Pro	Incremental cost-effectiveness ratio per QALY

#### 3.6.1. Types of investigations undertaken and metrics used

Studies in this category typically analysed the costs associated with scheduling, cancer treatment strategies, or new system-level interventions. Two papers addressed another problem type in addition to cost analysis (B. P. Berg et al., 2013; Rejeb et al., 2018). Metrics used in these papers varied from assessing the marginal costs per patient, the cost of treatments, the cost per life year saved, net gain, to the incremental cost-effectiveness ratio per quality-adjusted life years (QALYs). Studies commonly measured the QALYs alongside cost measurements.

#### 3.6.2. Care pathways studied

Papers that used DES followed the patient from patient presentation, to diagnostic tests/procedures, to recovery (B. P. Berg et al., 2013; Eldabi et al., 2000; Pilgrim et al., 2009). These papers typically investigated the impact on cost of a system-level intervention, e.g., B. P. Berg et al. (2013) evaluated the cost utility of patient no-show mitigation strategies. One cost analysis paper designed their discrete-event simulation to include a micro-costing analysis, and used this to determine the cost-effectiveness of health information systems on cancer treatments at a system level (Rejeb et al., 2018).

#### 3.6.3. Changes suggested

Changes suggested by papers were all at the organisational or system level, such as implementing new care programs (Round et al., 2014) or Health Informatics Systems (Rejeb et al., 2018), changing the organisation’s appointment overbooking policy (B. P. Berg et al., 2013), or changing general practitioner referral criteria (Pilgrim et al., 2009).

#### 3.6.4. Implementations

No papers mentioned their findings being implemented in practice, although one paper mentioned successfully conducting an initial pilot study with promising results (Romero et al., 2013). Another cost analysis paper stated their recommendations were proposed to senior management and were being considered for implementation at the time of writing (Santibáñez et al., 2009).

## 4. Discussion

We identified 51 papers that used computer simulation to support healthcare decision-making. Although relatively few papers described successful implementation, improvements reported included higher quality of life for patients (H. E. Carter et al., 2019), lower hospital costs (Masselink et al., 2012) and reduced clinician idle time/overtime (Santibáñez et al., 2009).

DES was the most used simulation tool, perhaps unsurprisingly given it can provide metrics on patient throughput time, waiting times, and enables modelling of complex systems. By describing the steps in a pathway as nodes of a network of queues, DES can be used to simulate patients moving from appointment to appointment. DES has been widely used to model care pathways and lends itself well to investigating policy changes/interventions (see Demir et al. (2018) and references within).

### 4.1. Limitations of this study

It should be noted that this review does not cover the whole domain of cancer services. Examples of simulation in healthcare systems that were done in-house or were not published would not have been included in our search. Additionally, we recognise that there may be additional literature outside English-language academic journals or not covered by the queried databases.

### 4.2. Engagement with stakeholders

Papers that mentioned operational engagement tended to involve clinicians at the start of the project, i.e., parameter estimation or designing the model and simulation, but much less at the end. Operational engagement has been shown to be critical for translating OR research into improvement of healthcare services (Boaz et al., 2015; Levasseur, 2010; Robinson & Pidd, 1998). But it is also lacking, particularly in the healthcare sector (Fone et al., 2003; Jahangirian et al., 2015; Taylor et al., 2009). This may be due to the difficulty of executing changes, or it may be that at the time of publishing the study no changes had yet been carried through. Monks (2015) expands on the importance of communication and cooperation between modellers and healthcare professionals to improve the usage of OR as an implementation science. This is reflected in our review which showed that 19 of the 33 studies that aimed to improve services in practice did not engage with clinicians or operational managers in more than one aspect of modelling. The papers that mentioned operational engagement overlapped with the papers that described successful implementations. Studies reporting a lack of or no engagement also did not describe implementing new policies or interventions (see Figure 5). This is consistent with previous findings (see Saville et al., 2019; Scheinker & Brandeau, 2017).

It has been suggested that this lack of engagement in simulation projects may be due to either lack of communication between the OR scientist and the clinicians, a lack of project support, or it may be that the clinical counterparts are too busy to devote the necessary amounts of time (Jahangirian et al., 2015). It may also be that the changes presented to key stakeholders were too technically complex or disruptive to workflows to implement (Scheinker & Brandeau, 2017). Engaging with clinicians requires additional work on the part of the modeller, which can extend the length of the project and, in some cases, a prolonged project might not be feasible or financially viable. Considering the pressure to publish results quickly, researchers may not wait to see whether their projects have been implemented before publishing results. It would be beneficial to see future papers published that reflect upon the outcomes of earlier projects.

Most of the successful implementation papers listed clinical colleagues or hospital counterparts as co-authors, suggesting a closer involvement with the collaborating hospital. Carter et al. suggests having a strong “champion” within the hospital who will lobby for the project and manage internal support and expectations (M. W. Carter & Busby, 2023). We focused this study on simulation projects that could drive a change in hospital operational decisions. Only 18 (35%) papers were conceptual or framework only studies. Perhaps surprisingly, we found that 24% of the remaining papers, 16% of all 51 papers, mentioned successful implementations. This is a higher percentage than reported in similar reviews about healthcare simulation in operational research, which have typically reported implementation rates of 2–5% (see Brailsford et al (2019, 2009); Katsaliaki and Mustafee (2011); Roy et al. (2021)). Our search strategy focused on problems related to hospital operational decisions, which may partly explain the higher implementation success rate.

The implemented projects provided hospital-specific suggestions, e.g., changes in appointment scheduling policies, a particular increase in capacity, or new drug preparation strategies. These changes would be carried out at a clinic or hospital level, potentially making it easier to implement than changes at the organisation or system level. Papers that did not report implementations typically recommended changes across multiple hospitals, did not list a clinical author, or were presenting new methodologies or algorithms. Many of the recommended changes involve increasing capacity, which can result in increased expenditures and may be a barrier to implementation.

### 4.3. Limitations of the papers

Papers mentioned a lack of data, or having access to only small data sets, or missing data (e.g., Lu et al. (2012); Santibáñez et al. (2009); Miranda and Miranda (2021); Lamé et al. (2020); Arafeh et al. (2014); Chalk et al. (2019); Pilgrim et al. (2009); Heller et al. (2012)). Lack of data risks inaccurate parameterisation of models which could lead to misleading conclusions. 10 (23%) papers involved healthcare experts to fill in the gaps left by the lack of data. Most of the studies were conducted at only one clinic or hospital, so the results may not be generalisable (e.g., see Kang and Haswell (2020)). One possible strategy for mitigating this limitation could be involving clinical teams in the project, particularly at the beginning when designing the model. Several took this approach and consulted clinical experts to obtain estimates of necessary input parameters (e.g., Ogulata et al. (2009); Lu et al. (2012); Coelli et al. (2007); Santibáñez et al. (2009)).

Cancer services can share resources with other services, which may not be true of emergency department/outpatient clinics who are the sole owners of their diagnostics. Cancer pathways typically have defined diagnostic services which may require multiple tests to happen in parallel or in sequence. DES, the most common simulation technique used, requires events to happen in sequential order. Waiting times for patients receiving multiple diagnostic tests in parallel would not be accurately reflected in the model. DES also assumes that each node along the pathway is an individual resource. However, cancer patients often have an outpatient appointment at a clinic, followed by a follow-up appointment at the same clinic, meaning two nodes would share a resource. Some authors addressed this by scaling their service rates to account for the fact that some resources are shared by other care pathways (Romero et al., 2013), however these solutions might not be sufficient to capture the complexity of the interplay between pathways.

Modelling cancer care can become overly complex if the modeller intends to capture patient behaviour, such as patient hesitancy towards engaging with treatments, or interactions between the cancer pathway and the rest of the hospital. The reviewed models often included multiple assumptions, which impact how well the results reflect real life. For example, some authors chose not to include patients who are late to their appointments or patients who drop out of the pathway. Some scheduling papers did not allow for patients to arrive late or not at all (van de Vrugt et al., 2017), did not account for no-show appointments (Ma et al., 2016; Ogulata et al., 2009), or assumed homogeneous patients (Burns et al., 2022). One study introduced a simulation tool that helps clinics analyse the trade-offs associated with colonoscopy scheduling policies (Martin et al., 2020). In the project, they used a scheduling policy to generate a schedule, which then fed into a DES and calculated waiting times and provider idle times and overtimes. However, their model assumed discrete patient times and did not consider other providers working in the system or the interactions between providers. Albeit often simplifications are simply necessary to create a useful model of cancer care, integrating more characteristics into the model would increase the accuracy and allow operational managers to make better informed decisions.

### 4.4. Future work

#### 4.4.1. Use hybrid simulations or expand upon existing simulation techniques

Only a few studies analysed in this review utilised techniques other than DES. Monte Carlo simulations were the second most common and were typically used for sensitivity analysis in resource allocation and cost analysis papers. Though often sufficient for modelling purposes, these techniques could be complemented by more sophisticated ones stepping up the level of insights achievable. For instance, agent-based modelling is useful for describing the interactions between agents and may be useful for analysing networks of cancer clinics (e.g., Carney et al. (2014)), assessing risk analysis tools (see Elliott et al. (2019) for a cancer screening example), or the behaviour of patients attending treatments at clinics. Also, augmenting DES models with patient characteristics may provide insights into how patients with multiple cancers progress through the care pathway. Similarly, an approach involving system dynamics could provide novel analysis for cancer services that may lead to better patient care (e.g., Gunecs et al., 2004; Hosking et al., 2013). For example, a system dynamics model can give a high-level view of policy changes and how a system’s behaviour changes in response to an intervention (Mielczarek, 2016).

**Table 9.** Suggestions for future work and potential scenarios where they may be useful.

Suggestion	Use-case
(1) Use hybrid simulations or expand upon existing simulation techniques	Model patient behaviour; decrease wait times across the system
(2) Model pathways specific to prevalent cancers, e.g., lung cancer	Focusing on specific improvements could increase chance of implementation
(3) Include the patient voice and patient demographics; patient engagement as well as operational	Improve quality of care received; address disparity in access to cancer care
(4) Investigate the viability of new technologies, e.g., Mobile outpatient units or telemedicine	Address disparity in access to cancer care; provide alternatives with the aim of decreasing wait times
(5) Model shared resources across the care pathway and between services	Address complex patient pathways and need for integrated care
(6) Utilise design of experiments techniques	Optimise workflows; provide user-friendly platforms for assessing the system

#### 4.4.2. Model other prevalent cancers, e.g., lung cancer

The most common cancer or treatment focuses were breast, colorectal, general cancer services, and chemotherapy, which likely reflects the fact that breast cancer is the most common cancer and chemotherapy the most common cancer treatment in the UK, and that colorectal cancer is the fourth highest cause of death from cancer worldwide (Favoriti et al., 2016). The NHS also identifies lung and prostate cancer as some of the most common types of cancers in the UK (Cancer Overview, 2022), yet only two reviewed papers were focused on improving lung cancer care services and studied prostate cancer services. It may be that the no papers were found on this topic, because the prostate cancer pathway is a simpler pathway and requires less optimisation from an operational standpoint. However, given the prevalence of these two cancers, this may be an area to concentrate on in the future (Maddams et al., 2012).

#### 4.4.3. Include the patient voice and patient demographics

Patient reported outcomes are the status of a patient’s condition and care journey as reported by the patient. They include outcomes that affect the patient’s experience, e.g., waiting times, number of hours spent in the hospital, or their probability of cancer survival. These outcomes can help improve the quality of cancer care for patients (LeBlanc & Abernethy, 2017). Only one paper mentioned involving the patients in their study, who used Six Sigma methodology to improve patient flow at an outpatient pharmacy at a cancer hospital (Arafeh et al., 2014). Researchers may want to consider incorporating the patient’s voice and engaging with not only their clinical counterparts, but with the patients themselves. One potential avenue for modelling this may be to use a combination of simulation techniques to model different levels of care simultaneously and capture both individual- and system-level characteristics (see van de Ven et al. (2022)).

Patient demographics are important for helping address the disparity in access to cancer care, an issue that has been exacerbated with the COVID-19 pandemic and affects a growing population of patients. Few papers considered the demographics of the patients in their system. Sullivan et al. argue that one of the barriers to providing affordable cancer care in high-income countries is an inequality in access to care (Sullivan et al., 2011). This may be a reason for modellers to consider different categories of patients (e.g., socioeconomic backgrounds, vulnerable patient groups) in their simulations and potential hospital-based changes (Penedo et al., 2020).

#### 4.4.4. Investigate the viability of new technologies

Alternatively, modellers should consider modelling alternative healthcare delivery solutions to determine their viability, such as telemedicine or eHealth platforms at community clinics (Penedo et al., 2020). In one study, the effect of a mobile radiation oncology system was evaluated to make oncology care more accessible to rural populations (Price et al., 2022), which usually have high rates of mortality from cancer (Levit et al., 2020).

#### 4.4.5. Model shared resources across the care pathway and between services

Patients with metastasised cancer, cancer that has progressed and spread, present with multiple co-morbidities, making their care much more complex and requiring care from many different services (Evans et al., 2015). Most papers modelled isolated systems or only looked at a part of the cancer pathway, rather than the system as whole. Research in the future may address the need for integrated care by extending existing DES techniques or using a system dynamics approach to analyse the interactions between cancer pathways.

#### 4.4.6. Utilise design of experiments techniques

Design of Experiment techniques were only used by one paper (Baril et al., 2020), but may provide an additional way of shedding light on how cancer systems behave. These statistical techniques are useful for studying the relationship between input and output variables. Especially in the absence of sufficient or reliable data, Design of Experiments could aid in parameterising and analysing the model. Additionally, the results of the experiments allow the modeller to create metamodels or response surfaces. These can be used to communicate system behaviour to stakeholders, or could even provider an interactive platform to be used in decision-making processes (for an example, see Hill et al., 2015).

## 7. Conclusion

We conducted a systematic review of the use of simulation techniques in cancer services. We present a categorisation and analysis of the publications within this field, which aims to provide direction for future work (Table 9). We particularly emphasise opportunities for exploiting the potential of state-of-the-art simulation techniques in relation to the specific characteristics of systems delivering cancer services. DES was the preferred simulation tool for all problem types so future research investigating other simulation techniques and their viability in cancer services could be of benefit. Projects often reported a lack of access to data sets, which Design of Experiment techniques may help with. Suggested changes were typically focused at the hospital-level and modelled specific parts of care the pathway or the provided services. Operational researchers addressing cancer care problems may want to consider these limitations if their aim is to implement changes to services. Future OR projects aiming to contribute to the implementation of service changes in practice may want to review the findings of this paper when designing their study.

We have highlighted where simulation projects within cancer care can improve, but it is important to note that all the papers studies in this review presented new decision-making tools or analysis that could potentially be of benefit to healthcare organisations.

## References

[cit0001] Ahmed, Z., ElMekkawy, T., & Bates, S. (2011). Developing an efficient scheduling template of a chemotherapy treatment unit: A case study. *The Australasian Medical Journal*, 4(10), 575. 10.4066/AMJ.2011.837 23386870 PMC3562880

[cit0002] Al-Abri, R., & Al-Balushi, A. (2014). Patient satisfaction survey as a tool towards quality improvement. *Oman Medical Journal*, 29(1), 3. 10.5001/omj.2014.02 24501659 PMC3910415

[cit0003] Almagooshi, S. (2015). Simulation modelling in healthcare: Challenges and trends. *Procedia Manufacturing*, 3, 301–307. 10.1016/j.promfg.2015.07.155

[cit0004] Alvarado, M. M., Cotton, T. G., Ntaimo, L., Perez, E., & Carpentier, W. R. (2018). Modeling and simulation of oncology clinic operations in discrete event system specification. *Simulation*, 94(2), 105–121. 10.1177/0037549717708246

[cit0005] Arafeh, M., Barghash, M. A., Sallam, E., & AlSamhouri, A. (2014). Six sigma applied to reduce patients’ waiting time in a cancer pharmacy. *International Journal of Six Sigma and Competitive Advantage*, 8(2), 105–124. 10.1504/IJSSCA.2014.064256

[cit0006] Aspland, E., Gartner, D., & Harper, P. (2021). Clinical pathway modelling: A literature review. *Health Systems*, 10(1), 1–23. 10.1080/20476965.2019.1652547 PMC7946019 33758656

[cit0007] Babashov, V., Aivas, I., Begen, M., Cao, J., Rodrigues, G., D’Souza, D., Lock, M., & Zaric, G. (2017). Reducing patient waiting times for radiation therapy and improving the treatment planning process: A discrete-event simulation model (radiation treatment planning). *Clinical Oncology*, 29(6), 385–391. 10.1016/j.clon.2017.01.039 28222957

[cit0008] Baril, C., Gascon, V., & Miller, J. (2020). Design of experiments and discrete-event simulation to study oncology nurse workload. *IISE Transactions on Healthcare Systems Engineering*, 10(1), 74–86. 10.1080/24725579.2019.1680581

[cit0009] Bauza, X., & Chow, J. C. (2019). An automated scheduling system for radiotherapy physicist on-call using monte carlo simulation. *Australasian Physical & Engineering Sciences in Medicine*, 42(1), 27–32. 10.1007/s13246-018-0705-0 30387002

[cit0010] Berg, B., Denton, B., Nelson, H., Balasubramanian, H., Rahman, A., Bailey, A., & Lindor, K. (2010). A discrete event simulation model to evaluate operational performance of a colonoscopy suite. *Medical Decision Making*, 30(3), 380–387. 10.1177/0272989X09345890 19773583

[cit0011] Berg, B. P., Murr, M., Chermak, D., Woodall, J., Pignone, M., Sandler, R. S., & Denton, B. T. (2013). Estimating the cost of no-shows and evaluating the effects of mitigation strategies. *Medical Decision Making*, 33(8), 976–985. 10.1177/0272989X13478194 23515215 PMC4153419

[cit0012] Bernatchou, M., Ouzayd, F., Bellabdaoui, A., & Hamdaoui, M. (2017). Towards a simulation model of an outpatient chemotherapy unit. In 2017 International Colloquium on Logistics and Supply Chain Management (LOGISTIQUA), Rabat, France, April 27-28, 2017 (pp. 177–182). IEEE.

[cit0013] Bespalov, A., Barchuk, A., Auvinen, A., & Nevalainen, J. (2021). Cancer screening simulation models: A state of the art review. *BMC Medical Informatics & Decision Making*, 21(1), 1–10. 10.1186/s12911-021-01713-5 34930233 PMC8690438

[cit0014] Blakely, T., Collinson, L., Kvizhinadze, G., Nair, N., Foster, R., Dennett, E., & Sarfati, D. (2015). Cancer care coordinators in stage iii colon cancer: a cost-utility analysis. *BMC Health Services Research*, 15(1), 1–12. 10.1186/s12913-015-0970-5 26238996 PMC4523949

[cit0015] Boaz, A., Hanney, S., Jones, T., & Soper, B. (2015). Does the engagement of clinicians and organisations in research improve healthcare performance: A three-stage review. *BMJ Open*, 5(12), e009415. 10.1136/bmjopen-2015-009415 PMC4680006 26656023

[cit0016] Boonmee, C., Kosayanon, A., Chitapanarux, I., & Kasemset, C. (2020). Radiotherapy service improvement: Simulation study. *Industrial Engineering & Management Systems*, 19(4), 758–773. 10.7232/iems.2020.19.4.758

[cit0017] Brailsford, S. C., Eldabi, T., Kunc, M., Mustafee, N., & Osorio, A. F. (2019). Hybrid simulation modelling in operational research: A state-of-the-art review. *European Journal of Operational Research*, 278(3), 721–737. 10.1016/j.ejor.2018.10.025

[cit0018] Brailsford, S. C., Harper, P. R., Patel, B., & Pitt, M. (2009). An analysis of the academic literature on simulation and modelling in health care. *Journal of Simulation*, 3(3), 130–140. 10.1057/jos.2009.10

[cit0019] Burns, P., Konda, S., & Alvarado, M. (2022). Discrete-event simulation and scheduling for mohs micrographic surgery. *Journal of Simulation*, 16(1), 43–57. 10.1080/17477778.2020.1750315

[cit0020] Camg ¨oz Akda ˘g, H., & Arsoy-Ilikan, D. (2018). Improvement of patient pathway in a breast cancer center. *IIOAB Journal*, 9(1), 16–26. 10.xxxx/iioabj.2018.1

[cit0021] Cancer overview. (2022). N. H. S. https://www.nhs.uk/conditions/cancer/.

[cit0022] Carney, T. J., Morgan, G. P., Jones, J., McDaniel, A. M., Weaver, M., Weiner, B., & Haggstrom, D. A. (2014). Using computational modeling to assess the impact of clinical decision support on cancer screening improvement strategies within the community health centers. *Journal of Biomedical Informatics*, 51, 200–209. 10.1016/j.jbi.2014.05.012 24953241 PMC4194243

[cit0023] Carter, M. W., & Busby, C. R. (2023). How can operational research make a real difference in healthcare? challenges of implementation. *European Journal of Operational Research*, 306(3), 1059–1068. 10.1016/j.ejor.2022.04.022

[cit0024] Carter, H. E., Knowles, D., Moroney, T., Holtmann, G., Rahman, T., Appleyard, M., Steele, N., Zanco, M., & Graves, N. (2019). The use of modelling studies to inform planning of health services: Case study of rapidly increasing endoscopy services in australia. *BMC Health Services Research*, 19(1), 1–8. 10.1186/s12913-019-4438-x 31464609 PMC6716875

[cit0025] Chalk, D., Trent, N., Vennam, S., McGrane, J., & Mantle, M. (2019). Reducing delays in the diagnosis and treatment of muscle-invasive bladder cancer using simulation modelling. *Journal of Clinical Urology*, 12(2), 129–133. 10.1177/2051415818794089

[cit0026] Chen-See, S. (2020). Disruption of cancer care in canada during covid-19. *The Lancet Oncology*, 21(8), e374. 10.1016/S1470-2045(20)30397-1 32711682 PMC7377791

[cit0027] Coelli, F. C., Ferreira, R. B., Almeida, R. M. V., & Pereira, W. C. A. (2007). Computer simulation and discrete-event models in the analysis of a mammography clinic patient flow. *Computer Methods and Programs in Biomedicine*, 87(3), 201–207. 10.1016/j.cmpb.2007.05.006 17606308

[cit0028] Coelli, F. C., Ferreira, R. B., Almeida, R. M., & Pereira, W. C. (2007). Computer simulation and discrete-event models in the analysis of a mammography clinic patient flow. *Computer Methods and Programs in Biomedicine*, 87(3), 201–207. 10.1016/j.cmpb.2007.05.006 17606308

[cit0029] Demir, E., Southern, D., Rashid, S., & Lebcir, R. (2018). A discrete event simulation model to evaluate the treatment pathways of patients with cataract in the united kingdom. *BMC Health Services Research*, 18(1), 1–15. 10.1186/s12913-018-3741-2 30514277 PMC6278024

[cit0030] Edge, R., Meyers, J., Tiernan, G., Li, Z., Schiavuzzi, A., Chan, P., Vassallo, A., Morrow, A., Mazariego, C., Wakefield, C. E., Canfell, K., & Taylor, N. (2021). Cancer care disruption and reorganisation during the COVID-19 pandemic in australia: A patient, carer and healthcare worker perspective. *Public Library of Science ONE*, 16(9), e0257420. 10.1371/journal.pone.0257420 34534231 PMC8448370

[cit0031] Eldabi, T., Paul, R. J., & Taylor, S. J. (2000). Simulating economic factors in adjuvant breast cancer treatment. *Journal of the Operational Research Society*, 51(4), 465–475. 10.1057/palgrave.jors.2600881

[cit0032] Elliott, T. M., Lord, A., Simms, L. A., Radford-Smith, G., Valery, P. C., & Gordon, L. G. (2019). Evaluating a risk assessment tool to improve triaging of patients to colonoscopies. *Internal Medicine Journal*, 49(10), 1292–1299. 10.1111/imj.14267 30816603

[cit0033] England, T. J., Harper, P. R., Crosby, T., Gartner, D., Arruda, E. F., Foley, K. G., & Williamson, I. J. (2021). Examining the diagnostic pathway for lung cancer patients in wales using discrete event simulation. *Translational Lung Cancer Research*, 10(3), 1368. 10.21037/tlcr-20-919 33889516 PMC8044476

[cit0034] England, T., Harper, P., Crosby, T., Gartner, D., Arruda, E. F., Foley, K., & Williamson, I. (2021). Modelling lung cancer diagnostic pathways using discrete event simulation. *Journal of Simulation*, 17(1), 94–104. 10.1080/17477778.2021.1956866 36760877 PMC9901409

[cit0035] Evans, J. M., Matheson, G., Buchman, S., MacKinnon, M., Meertens, E., Ross, J., & Johal, H. (2015). Integrating cancer care beyond the hospital and across the cancer pathway: A patient-centred approach. *Healthcare Quarterly*, 17(SP), 28–32. 10.12927/hcq.2014.24006 25562131

[cit0036] Favoriti, P., Carbone, G., Greco, M., Pirozzi, F., Pirozzi, R. E. M., & Corcione, F. (2016). Worldwide burden of colorectal cancer: A review. *Updates in Surgery*, 68(1), 7–11. 10.1007/s13304-016-0359-y 27067591

[cit0037] Fetter, R. B., & Thompson, J. D. (1965). The simulation of hospital systems. *Operations Research*, 13(5), 689–711. 10.1287/opre.13.5.689

[cit0038] Fone, D., Hollinghurst, S., Temple, M., Round, A., Lester, N., Weightman, A., Roberts, K., Coyle, E., Bevan, G., & Palmer, S. (2003). Systematic review of the use and value of computer sim ulation modelling in population health and health care delivery. *Journal of Public Health*, 25(4), 325–335. 10.1093/pubmed/fdg075 14747592

[cit0039] Gough, D., Thomas, J., & Oliver, S. (2012). Clarifying differences between review designs and methods. *Systematic Reviews*, 1(1), 1–9. 10.1186/2046-4053-1-28 22681772 PMC3533815

[cit0040] Gunecs, E. D., Chick, S. E., & Aksin, O. Z. (2004). Breast cancer screening services: Trade-offs in quality, capacity, outreach, and centralization. *Health Care Management Science*, 7(4), 291–303. 10.1007/s10729-004-7538-y 15717814

[cit0041] Hadid, M., Elomri, A., Padmanabhan, R., Kerbache, L., Jouini, O., El Omri, A., Nounou, A., & Hamad, A. (2022). Clustering and stochastic simulation optimization for outpatient chemotherapy appointment planning and scheduling. *International Journal of Environmental Research and Public Health*, 19(23), 15539. 10.3390/ijerph192315539 36497611 PMC9736607

[cit0042] Heller, M., Zanocco, K., Zydowicz, S., Elaraj, D., Nayar, R., & Sturgeon, C. (2012). Cost-effectiveness analysis of repeat fine-needle aspiration for thyroid biopsies read as atypia of undetermined significance. *Surgery*, 152(3), 423–430. 10.1016/j.surg.2012.05.038 22938902

[cit0043] Heshmat, M., & Eltawil, A. (2021). Solving operational problems in outpatient chemotherapy clinics using mathematical programming and simulation. *Annals of Operations Research*, 298(1), 289–306. 10.1007/s10479-019-03500-y

[cit0044] Hill, R. R., Gutman, A. J., Moulder, R. D., Stafford, T. D., & Bush, K. R. (2015). Case studies: Definitive screening applied to a simulation study of the f100-229 engine repair network. *Quality Engineering*, 27(4), 424–436. 10.1080/08982112.2015.1023315

[cit0045] Holm, L. B., Bjornenak, T., Kjaeserud, G. G., & Noddeland, H. (2017). Using discrete event simulation and soft systems methodology for optimizing patient flow and resource utilization at the surgical unit of radiumhospitalet in oslo, norway. In 2017 Winter Simulation Conference (WSC), Las Vegas, Nevada, USA, December 3-6, 2017 (pp. 1646–1657). IEEE.

[cit0046] Hosking, M., Roberts, S., Uzsoy, R., & Joseph, T. M. (2013). Investigating interventions for increasing colorectal cancer screening: Insights from a simulation model. *Socio-Economic Planning Sciences*, 47(2), 142–155. 10.1016/j.seps.2012.10.001

[cit0047] Jahangirian, M., Taylor, S. J., Eatock, J., Stergioulas, L. K., & Taylor, P. M. (2015). Causal study of low stakeholder engagement in healthcare simulation projects. *Journal of the Operational Research Society*, 66(3), 369–379. 10.1057/jors.2014.1

[cit0048] Kang, H., & Haswell, E. (2020). Patient flow analysis using real-time locating system data: A case study in an outpatient oncology center. *JCO Oncology Practice*, 16(12), e1471–e1480. 10.1200/OP.20.00119 32628564

[cit0049] Katsaliaki, K., & Mustafee, N. (2011). Applications of simulation within the healthcare context. *Journal of the Operational Research Society*, 62(8), 1431–1451. 10.1057/jors.2010.20 32226177 PMC7099916

[cit0050] Kazemian, P., Sir, M. Y., Van Oyen, M. P., Lovely, J. K., Larson, D. W., & Pasupathy, K. S. (2017). Coordinating clinic and surgery appointments to meet access service levels for elective surgery. *Journal of Biomedical Informatics*, 66, 105–115. 10.1016/j.jbi.2016.11.007 27993748

[cit0051] Keshtzari, M., & Norman, B. A. (2022). Improving patient access in oncology clinics using simulation. *Journal of Industrial Engineering & Management*, 15(3), 455–469. 10.3926/jiem.3925

[cit0052] Lamé, G., Jouini, O., & Stal Le Cardinal, J. (oct. 2020). Combining soft systems methodology, ethnographic observation, and discrete-event simulation: A case study in cancer care. *Journal of the Operational Research Society*, 71(10), 1545–1562. 10.1080/01605682.2019.1610339

[cit0053] Laviana, A. A., Luckenbaugh, A. N., & Resnick, M. J. (2020). Trends in the cost of cancer care: Beyond drugs. *Journal of Clinical Oncology*, 38(4), 316. 10.1200/JCO.19.01963 31804864 PMC6994251

[cit0054] LeBlanc, T. W., & Abernethy, A. P. (2017). Patient-reported outcomes in cancer care—hearing the patient voice at greater volume. *Nature Reviews Clinical Oncology*, 14(12), 763–772. 10.1038/nrclinonc.2017.153 28975931

[cit0055] Levasseur, R. E. (2010). People skills: Ensuring project success—a change management perspective. *Interfaces*, 40(2), 159–162. 10.1287/inte.1090.0473

[cit0056] Levit, L. A., Byatt, L., Lyss, A. P., Paskett, E. D., Levit, K., Kirkwood, K., Schenkel, C., & Schilsky, R. L. (2020). Closing the rural cancer care gap: Three institutional approaches. *JCO Oncology Practice*, 16(7), 422–430. 10.1200/OP.20.00174 32574128

[cit0057] Liu, E., Ma, X., Saure, A., Weber, L., Puterman, M. L., & Tyldesley, S. (2019). Improving access to chemotherapy through enhanced capacity planning and patient scheduling. *IISE Transactions on Healthcare Systems Engineering*, 9(1), 1–13. 10.1080/24725579.2018.1442376

[cit0058] Luengo-Fernandez, R., Leal, J., Gray, A., & Sullivan, R. (2013). Economic burden of cancer across the european union: A population-based cost analysis. *The Lancet Oncology*, 14(12), 1165–1174. 10.1016/S1470-2045(13)70442-X 24131614

[cit0059] Lu, T., Wang, S., Li, J., Lucas, P., Anderson, M., & Ross, K. (2012). A simulation study to improve performance in the preparation and delivery of antineoplastic medications at a community hospital. *Journal of Medical Systems*, 36(5), 3083–3089. 10.1007/s10916-011-9786-y 22072277

[cit0060] Maddams, J., Utley, M., & Møller, H. (2012). Projections of cancer prevalence in the united kingdom, 2010–2040. *British Journal of Cancer*, 107(7), 1195–1202. 10.1038/bjc.2012.366 22892390 PMC3461160

[cit0061] Mariotto, A. B., Enewold, L., Zhao, J., Zeruto, C. A., & Yabroff, K. R. (2020). Medical care costs associated with cancer survivorship in the united states. *Cancer Epidemiology, Biomarkers & Prevention*, 29(7), 1304–1312. 10.1158/1055-9965.EPI-19-1534 PMC9514601 32522832

[cit0062] Martin, J., Singh, P., Cohn, A., Kiel-Locey, J., Shehadeh, K., Saini, S. D., & Kurlander, J. E. (2020). Integrated simulation tool to analyze patient access to and flow during colonoscopy appointments. In 2020 Winter Simulation Conference (WSC), Orlando, Florida, USA, December 14-18, 2020 (pp. 934–943). IEEE.

[cit0063] Ma, X., Saure, A., Puterman, M. L., Taylor, M., & Tyldesley, S. (2016). Capacity planning and appointment scheduling for new patient oncology consults. *Health Care Management Science*, 19(4), 347–361. 10.1007/s10729-015-9331-5 26156688

[cit0064] Masselink, I. H., van der Mijden, T. L., Litvak, N., & Vanberkel, P. T. (2012). Preparation of chemotherapy drugs: Planning policy for reduced waiting times. *Omega*, 40(2), 181–187. 10.1016/j.omega.2011.05.003

[cit0065] McKinley, K. W., Babineau, J., Roskind, C. G., Sonnett, M., & Doan, Q. (2020). Discrete event simulation modelling to evaluate the impact of a quality improvement initiative on patient flow in a paediatric emergency department. *Emergency Medicine Journal*, 37(4), 193–199. 10.1136/emermed-2019-208667 31915264

[cit0066] Mielczarek, B. (2016). Review of modelling approaches for healthcare simulation. *Operations Research and Decisions*, 26(1), 55–72.

[cit0067] Miranda, D. M. M., & Miranda, M. M. P. M. (2021). Discrete-event simulation applied to a radiotherapy process: A case study of a cancer center. *Brazilian Journal of Operations & Production Management*, 18(1), 1–20. 10.14488/BJOPM.2021.012

[cit0068] Monks, T. (2015). Operational research as implementation science: Definitions, challenges and research priorities. *Implementation Science*, 11(1), 1–10. 10.1186/s13012-016-0444-0 PMC4895878 27268021

[cit0069] Monks, T., Currie, C. S., Onggo, B. S., Robinson, S., Kunc, M., & Taylor, S. J. (2019). Strengthening the reporting of empirical simulation studies: Introducing the stress guidelines. *Journal of Simulation*, 13(1), 55–67. 10.1080/17477778.2018.1442155

[cit0070] Neal, R., Tharmanathan, P., France, B., Din, N., Cotton, S., Fallon-Ferguson, J., Hamilton, W., Hendry, A., Hendry, M., Lewis, R., Macleod, U., Mitchell, E. D., Pickett, M., Rai, T., Shaw, K., Stuart, N., Tørring, M. L., Wilkinson, C., Williams, B., Williams, N., & Williams, N. (2015). Is increased time to diagnosis and treatment in symptomatic cancer associated with poorer outcomes? systematic review. *British Journal of Cancer*, 112(1), S92–S107. 10.1038/bjc.2015.48 25734382 PMC4385982

[cit0071] NHS. (2022). Delivering cancer wait times: A good practice guide. https://www.england.nhs.uk/wp-content/uploads/2015/03/delivering-cancer-wait-times.pdf.

[cit0072] Ogulata, S. N., Cetik, M. O., Koyuncu, E., & Koyuncu, M. (2009). A simulation approach for scheduling patients in the department of radiation oncology. *Journal of Medical Systems*, 33(3), 233–239. 10.1007/s10916-008-9184-2 19408457

[cit0073] Palmer, R., Fulop, N. J., & Utley, M. (2017). A systematic literature review of operational researchmethods for modelling patient flow and outcomes within community healthcare and other settings. *Health Systems*, 7(1), 29–50. 10.1057/s41306-017-0024-9 PMC6452842 31214337

[cit0074] Patt, D., Gordan, L., Diaz, M., Okon, T., Grady, L., Harmison, M., Markward, N., Sullivan, M., Peng, J., & Zhou, A. (2020). Impact of COVID-19 on cancer care: How the pandemic is delaying cancer diagnosis and treatment for american seniors. *JCO Clinical Cancer Informatics*, 4(4), 1059–1071. 10.1200/CCI.20.00134 33253013 PMC7713534

[cit0075] Penedo, F. J., Oswald, L. B., Kronenfeld, J. P., Garcia, S. F., Cella, D., & Yanez, B. (2020). The increasing value of ehealth in the delivery of patient-centred cancer care. *The Lancet Oncology*, 21(5), e240–e251. 10.1016/S1470-2045(20)30021-8 32359500 PMC7643123

[cit0076] Pereira, G., Dias, L., Vik, P., & Oliveira, J. A. (2011). Discrete simulation tools ranking: A commercial software packages comparison based on popularity. In CAlg - Artigos em livros de atas/Papers in proceedings, Industrial Simulation Conference 2011, Centro Culturale Don Orione, Venice, Italy, June 6-8, 2011. EUROSIS-ETI.

[cit0077] Pilgrim, H., Tappenden, P., Chilcott, J., Bending, M., Trueman, P., Shorthouse, A., & Tappenden, J. (2009). The costs and benefits of bowel cancer service developments using discrete event simulation. *The Journal of the Operational Research Society*, 60(10), 1305–1314. 10.1057/jors.2008.109

[cit0078] Price, A. T., Canfield, C., Hugo, G. D., Kavanaugh, J. A., Henke, L. E., Laugeman, E., Samson, P., Reynolds-Kueny, C., & Cudney, E. A. (2022). Techno-economic feasibility analysis of a fully mobile radiation oncology system using monte carlo simulation. *JCO Global Oncology*, 8(8), e2100284. 10.1200/GO.21.00284 35609229 PMC9173580

[cit0079] Rejeb, O., Pilet, C., Hamana, S., Xie, X., Durand, T., Aloui, S., Doly, A., Biron, P., Perrier, L., & Augusto, V. (2018). Performance and cost evaluation of health information systems using micro-costing and discrete-event simulation. *Health Care Management Science*, 21(2), 204–223. 10.1007/s10729-017-9402-x 28516345

[cit0080] Richards, M., Anderson, M., Carter, P., Ebert, B. L., & Mossialos, E. (2020). The impact of thecovid-19 pandemic on cancer care. *Nature Cancer*, 1(6), 565–567. 10.1038/s43018-020-0074-y 35121972 PMC7238956

[cit0081] Robinson, S., & Pidd, M. (1998). Provider and customer expectations of successful simulationprojects. *Journal of the Operational Research Society*, 49(3), 200–209. 10.1057/palgrave.jors.2600516

[cit0082] Romero, H., Dellaert, N., Van Der Geer, S., Frunt, M., Jansen-Vullers, M., & Krekels, G. (2013). Admission and capacity planning for the implementation of one-stop-shop in skin cancer treatment using simulation-based optimization. *Health Care Management Science*, 16(1), 75–86. 10.1007/s10729-012-9213-z 22961383

[cit0083] Round, J., Leurent, B., & Jones, L. (2014). A cost-utility analysis of a rehabilitation service for people living with and beyond cancer. *BMC Health Services Research*, 14(1), 1–11. 10.1186/s12913-014-0558-5 25407558 PMC4245741

[cit0084] Roy, S., Prasanna Venkatesan, S., & Goh, M. (2021). Healthcare services: A systematic review ofpatient-centric logistics issues using simulation. *The Journal of the Operational Research Society*, 72(10), 2342–2364. 10.1080/01605682.2020.1790306

[cit0085] Santibáñez, P., Chow, V. S., French, J., Puterman, M. L., & Tyldesley, S. (2009). Reducing patient wait times and improving resource utilization at british columbia cancer agency’s ambulatory care unit through simulation. *Health Care Management Science*, 12(4), 392–407. 10.1007/s10729-009-9103-1 20058528

[cit0086] Saville, C. E., Smith, H. K., & Bijak, K. (2019). Operational research techniques applied throughoutcancer care services: a review. *Health Systems*, 8(1), 52–73. 10.1080/20476965.2017.1414741 31214354 PMC6507866

[cit0087] Scheinker, D., & Brandeau, M. L. (2017). Implementing analytics projects in a hospital: Successes, Failures, and opportunities. *INFORMS Journal on Applied Analytics*, 50(3), 176–189. 10.1287/inte.2020.1036

[cit0088] Singh, A. R., Gupta, A., Satpathy, S., & Gowda, N. (2022). Study to assess the utility of discrete event simulation software in projection & optimization of resources in the out-patient department at an apex cancer institute in india. *Health Science Reports*, 5(3), e627. 10.1002/hsr2.627 35509391 PMC9059176

[cit0089] Slocum, R. F., Jones, H. L., Fletcher, M. T., McConnell, B. M., Hodgson, T. J., Taheri, J., & Wilson, J. R. (2021). Improving chemotherapy infusion operations through the simulation of scheduling heuristics: A case study. *Health Systems*, 10(3), 163–178. 10.1080/20476965.2019.1709908 34377441 PMC8330715

[cit0090] Smith, A. F., Frempong, S. N., Sharma, N., Neal, R. D., Hick, L., & Shinkins, B. (2022). An exploratory assessment of the impact of a novel risk assessment test on breast cancer clinic waiting times and workflow: A discrete event simulation model. *BMC Health Services Research*, 22(1), 1301. 10.1186/s12913-022-08665-0 36309678 PMC9617530

[cit0091] Sullivan, R., Peppercorn, J., Sikora, K., Zalcberg, J., Meropol, N. J., Amir, E., Khayat, D., Boyle, P., Autier, P., Tannock, I. F., Fojo, T., Siderov, J., Williamson, S., Camporesi, S., McVie, J. G., Purushotham, A. D., Naredi, P., Eggermont, A., Brennan, M. F., Steinberg, M. L., & Kerr, D. (2011). Delivering affordable cancer care in high-income countries. *The Lancet Oncology*, 12(10), 933–980. 10.1016/S1470-2045(11)70141-3 21958503

[cit0092] Suss, S., Bhuiyan, N., Demirli, K., & Batist, G. (2018). Achieving level patient flow in an outpatientoncology clinic. *IISE Transactions on Healthcare Systems Engineering*, 8(1), 47–58. 10.1080/24725579.2017.1403521

[cit0093] Taylor, S. J., Eldabi, T., Riley, G., Paul, R. J., & Pidd, M. (2009). Simulation modelling is 50! do weneed a reality check? *Journal of the Operational Research Society*, 60(1), S69–S82. 10.1057/jors.2008.196

[cit0094] Thomas, S. J. (2003). Capacity and demand models for radiotherapy treatment machines. *Clinical Oncology*, 15(6), 353–358. 10.1016/S0936-6555(03)00065-7 14524490

[cit0095] Thomas, S., Williams, M., Burnet, N., & Baker, C. (2001). How much surplus capacity is required tomaintain low waiting times? *Clinical Oncology*, 13(1), 24–28. 10.1053/clon.2001.9210 11292132

[cit0096] van de Ven, M., IJzerman, M., Retèl, V., van Harten, W., & Koffijberg, H. (2022). Developing a dynamic simulation model to support the nationwide implementation of whole genome sequencing in lung cancer. *BMC Medical Research Methodology*, 22(1), 1–12. 10.1186/s12874-022-01571-3 35350994 PMC8962015

[cit0097] van de Vrugt, M., Boucherie, R. J., Smilde, T. J., de Jong, M., & Bessems, M. (2017). Rapid diagnoses at the breast center of jeroen bosch hospital: A case study invoking queueing theory and discrete event simulation. *Health Systems*, 6(1), 77–89. 10.1057/s41306-016-0013-4

[cit0098] Vieira, B., Demirtas, D., van de Kamer, J. B., Hans, E. W., & Van Harten, W. (2019). Improvingworkflow control in radiotherapy using discrete-event simulation. *BMC Medical Informatics and Decision Making*, 19(1), 1–13. 10.1186/s12911-019-0910-0 31651304 PMC6814107

[cit0099] Walker, C., O’Sullivan, M., Ziedins, I., & Furian, N. (2016). Faster cancer treatment: Using timestamp data to improve patient journeys. *Healthcare*, 4(4), 252–258. 10.1016/j.hjdsi.2016.04.012 28007222

[cit0100] Werker, G., Saure, A., French, J., & Shechter, S. (2009). The use of discrete-event simulation modelling to improve radiation therapy planning processes. *Radiotherapy and Oncology*, 92(1), 76–82. 10.1016/j.radonc.2009.03.012 19356818

[cit0101] World Cancer Research Fund. (2023). UK Cancer Statistics. https://www.wcrf-uk.org/preventing-cancer/uk-cancer-statistics/.

